# *Candida parapsilosis* Biofilm Identification by Raman Spectroscopy

**DOI:** 10.3390/ijms151223924

**Published:** 2014-12-22

**Authors:** Ota Samek, Katarina Mlynariková, Silvie Bernatová, Jan Ježek, Vladislav Krzyžánek, Martin Šiler, Pavel Zemánek, Filip Růžička, Veronika Holá, Martina Mahelová

**Affiliations:** 1 Institute of Scientific Instruments of the Academy of Sciences of the Czech Republic, v.v.i., Královopolská 147, Brno 61264, Czech Republic; E-Mails: berns@isibrno.cz (S.B.); jezek@isibrno.cz (J.J.); vlk@isibrno.cz (V.K.); siler@isibrno.cz (M.S); pavlik@isibrno.cz (P.Z.); 2 Department of Microbiology, Faculty of Medicine and St. Anne’s Faculty Hospital, Brno 65691, Czech Republic; E-Mails: k.mlynarikova@gmail.com (K.M.); fruzic@fnusa.cz (F.R.); veronika.hola@fnusa.cz (V.H.); martina.mahelova@fnusa.cz (M.M.)

**Keywords:** Raman spectroscopy, Candida parapsilosis, biofilm

## Abstract

Colonies of *Candida parapsilosis* on culture plates were probed directly *in situ* using Raman spectroscopy for rapid identification of specific strains separated by a given time intervals (up to months apart). To classify the Raman spectra, data analysis was performed using the approach of principal component analysis (PCA). The analysis of the data sets generated during the scans of individual colonies reveals that despite the inhomogeneity of the biological samples unambiguous associations to individual strains (two biofilm-positive and two biofilm-negative) could be made.

## 1. Introduction

When characterizing yeast colonies or biofilms using spectroscopic techniques, and specifically Raman spectroscopy, one is normally faced with the problem of spatial inhomogeneity of the sample. On one hand, this allows one to evaluate the response of a bio-organism to slightly different environmental conditions, as a function of position, but on the other hand it might hinder the clear identification of a particular biological specimen/molecular compound within a complex spectrum.

Regardless of this complication, it has been shown that the technique of Raman spectroscopy (including Raman imaging) can be regarded as the method of choice for many studies of micro-organisms, cells and biological samples [1,2,3,4,5,6,7,8,9,10,11,12,13]. A recent review provides valuable information on Raman spectroscopy in biomedicine for the characterization of molecular complexes in living cells and tissues [7]. In addition, a reasonably detailed database of Raman features encountered in biological samples was published [5]. For the compositional analysis and the spatial visualization of microbial colonies or biofilms, experiments have recently been performed in which spectra were acquired point-by-point, at a few selected positions of individual colonies [12,13]. Both particular species and some relevant molecular complexes could be identified this way, with some spatial resolution. This type of spot investigation for species and/or compound identification collect sufficient or complete (multi-spectra) information about the whole colony and respective the likely inhomogeneous growth of micro-organisms over the dimension of the sample.

We would like to note that because of a typical convex shape of a mature yeast colony (the colony height/elevation can be in order of hundreds of µm) it is difficult to apply commercial line-scan techniques (because of problems with re-focusing on the steep colony surface) where building up a spatial map can be achieved over relatively short time intervals. Near the periphery of a growing colony, the height rises steeply to a ridge and beyond the point of inflexion marking this ridge, the height rises less steeply to a flat center [14]. Thus, a possible solution to reliably apply Raman technique when large height differences between the periphery of a colony and its centre are introduced is spot measurement. In this way, a compositional map is build up from the significant part of a colony.

Building up of a spatial map can be achieved over relatively short time intervals; for example, yeast colonies can be analyzed in a few minutes for analysis and identification using standard chemometric techniques.

The investigation presented here expands on the findings from our earlier publication on Raman spectroscopy of bacterial strains, directly measured within the environment of the cultivation medium [8,9,10] in which we explored the ability of Raman spectroscopy for discriminating *S. epidermidis* to the level of different bacterial strains.

In the present study, we exploit point-by-point recording for significant parts of yeast colonies for each strain. Specifically, we restricted the spot measurements to the central, middle and upper periphery of the colony surfaces with appropriate refocusing on the sample for each Raman spectra to stay within the focal depth of the laser excitation and imaging optics. In this way, biological heterogeneity of a particular colony could be measured and introduced to the Raman spectra, visualized and analyzed.

**Figure 1:**
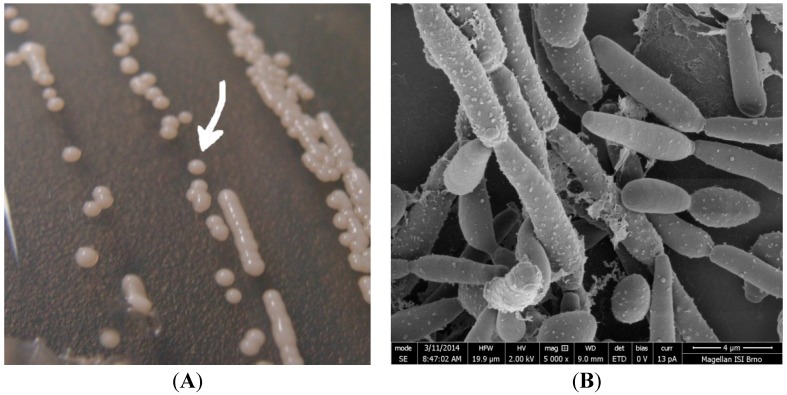
(**A**) Image of *Candida parapsilosis* colonies cultivated on agar for 48 h (the arrow shows selected colony for Raman analysis). The colony size is about 2 mm; (**B**) SEM (Scanning electron microscopy) image of *Candida parapsilosis* (cultivation for 48 h on a glass substrate), detailed image was obtained using combined preparation of the two techniques—chemical fixation and freeze drying (using ACE600 Leica microsystems).

We have analyzed colonies produced by four yeast strains identified as *Candida parapsilosis* (two biofilm-positive and two biofilm-negative) to evaluate the potential of Raman spectroscopy (Figure 1). Thus, distinction between biofilm positive and biofilm negative strains of the yeasts and the reproducibility of the measurement can be evaluated. Using the data sets, biofilm positive and negative strains could be unambiguously identified using principal component analysis (PCA).

## 2. Results and Discussion

**Figure 2:**
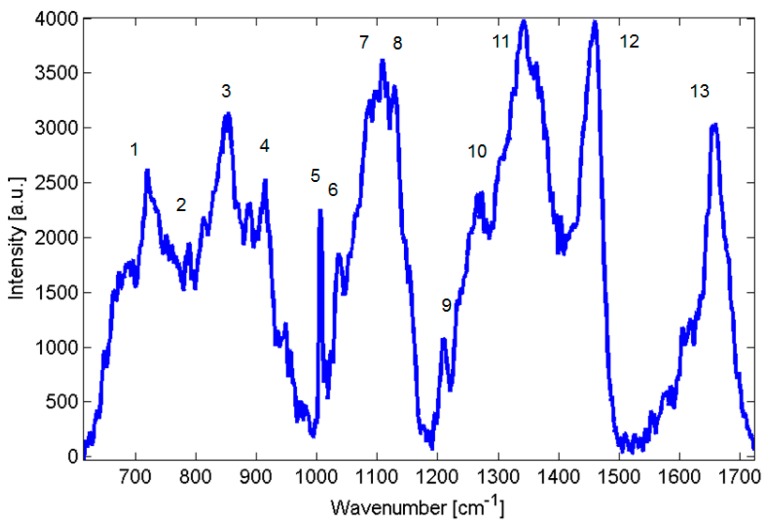
Raman spectra of *Candida parapsilosis.*

**Figure 3:**
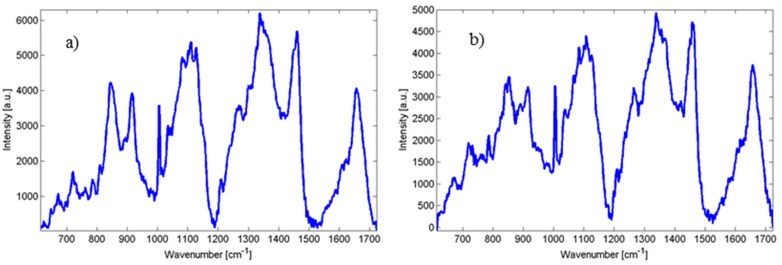
(**a**) Raman spectra of Candida orthopsilosis; and (**b**) Candida metapsilosis.

As mentioned further in experimental section, in order to assess the reproducibility of Raman spectroscopy, we inoculated MH agar with four *Candida parapsilosis* strains—BC11, BC16, BC45 and BC90 and, consequently, collected data from a minimum of 3 different colonies averaging at least three to four different points on each colony using the Renishaw inVia Raman system. Raw Raman spectra are shown in Figure 2, suggested assignment of lines is presented in Table 1. For comparison, in Figure 3 two closely related species of *Candida orthopsilosis* and *Candida metapsilosis* are shown. The incidence of fungal infection due to these species has increased in the last years and these species have not been widely explored using Raman spectroscopy.

**Table 1:** Summary of prominent peaks/bands observed in the Raman spectra of yeast, together with suggested assignments of chemical compounds. The peak numbers of the table are used to identify features in the spectra shown at Figure 2.

Peak No.	Raman Feature, cm^−1^	Suggested Assignment [3,8,9,10,11]
1	720	Adenine
2	782–788	782 cytosine, uracil, thymine. ring breathing
		788 O-P-O stretch of DNA
3	813	O-P-O stretch of RNA
4	880	C-C-N symmetric stretch of lipids
5	1002	Symmetric-ring breathing of phenylalanine amino acid
6	1033	C-H in-plane stretch of Phe
7	1080–1095	1080 C-C stretch of lipids
		1093 C-N stretch of proteins
		1095 vibration of phosphor dioxy (PO_2_) group
8	1128	1128 C-N stretch of proteins
9	1209	Proteins
10	1267–1270	Lipids, Amide III
11	1340–1350	Proteins, Carbohydrates
12	1440–1460	Deformation vibration CH_2_ scissoring, Lipids
13	1660–1670	Amide I, Lipids

**Figure 4:**
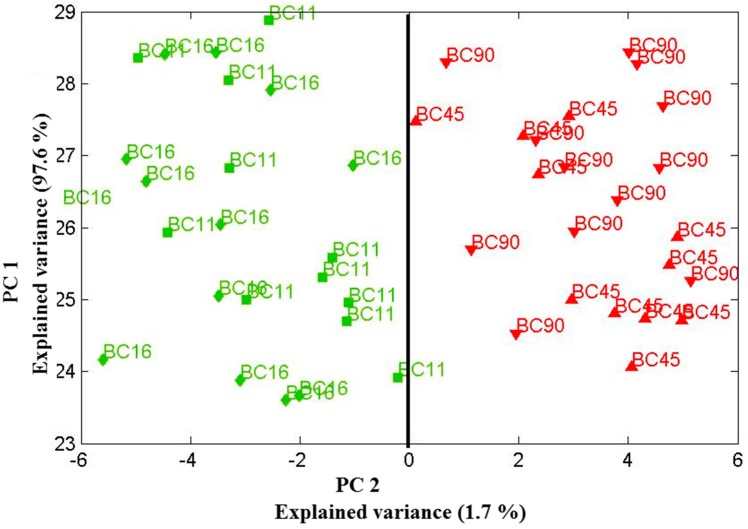
Scores plot of the first two principal components relation for four *Candida parapsilosis* strains (BC 11, BC 16, BC 45 and BC 90) cultured for 48 h. Using the two principle components, one can clearly separate the clusters of spectra associated with the biofilm-positive (BC 11, BC 16) and biofilm-negative (BC 45, BC 90) strains. Data sets were recorded on 30 July 2013.

**Figure 5:**
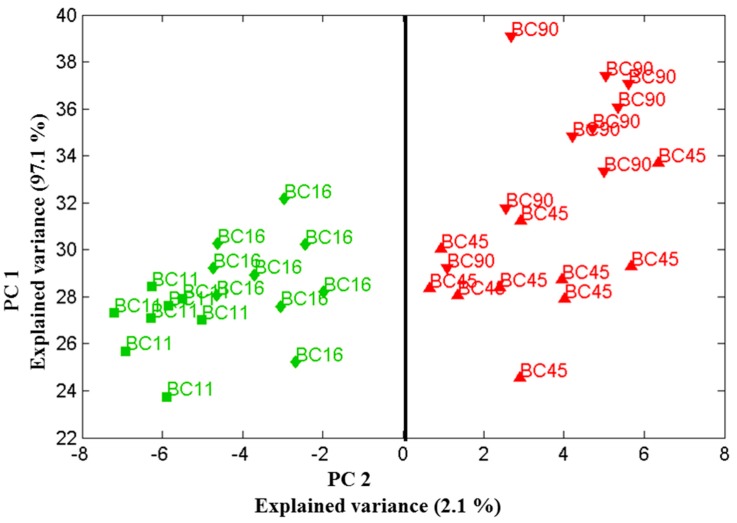
Scores plot of the first two principal components relation for four *Candida parapsilosis* strains (BC 11, BC 16, BC 45 and BC 90) cultured for 48 h. Using the two principle components, one can clearly separate the clusters of spectra associated with the biofilm-positive (BC 11, BC 16) and biofilm-negative (BC 45, BC 90) strains. Data sets were recorded on 19 December 2013.

**Figure 6:**
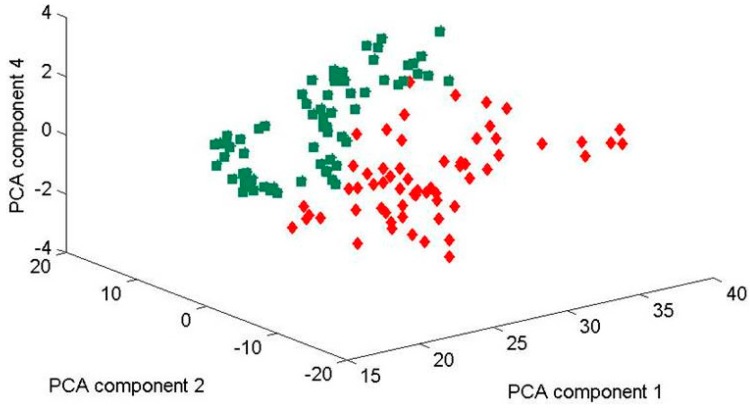
3-D-scores plot of principle component relation (PC1, PC2, and PC4) for four *Candida parapsilosis* strains (BC 11, BC 16, BC 45 and BC 90) cultured for 48 h. Using the three principle components, one can clearly separate the clusters of spectra associated with the biofilm-positive (BC 11, BC 16) and biofilm-negative (BC 45, BC 90) strains. Green data sets (on the left) and red (on the right side) include all the data measured at three different days (30 July, 19 December 2013 and 13 February 2014). Inspecting this data show that the clustering of the data sets—for two-biofilm positive and two biofilm-negative strains can be separated by the two clear clusters, although the first and the last data sets were recorded about six months apart (July 2013 and February 2014). This demonstrates the solid reproducibility in the Raman fingerprints of these biofilm-positive and negative strains. Explained variances of PC1 (97%), PC2 (2%), and PC4 (0.5%).

Applied chemometric principal component analysis of these spectra sets generated clusters of data points, from which the reproducibility of the measurement could be analyzed. Data were recorded in three different days of measurement (Figure 4, Figure 5 and Figure 6. This type of data could be used to compare yeast at both the species and strain level and allowed us to investigate the influence of successive principal components on the ability to differentiate between biofilm-positive and biofilm-negative strains.

**Figure 7:**
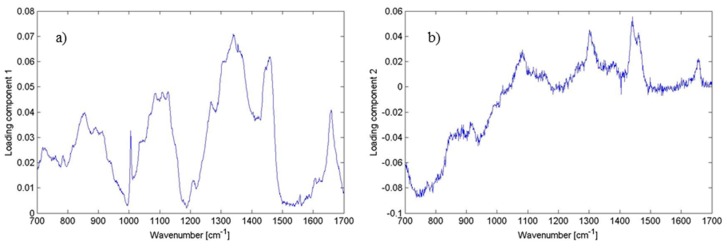
Plot of loadings of (**a**) PC1; and (**b**) PC2 corresponding to Figure 5. Different features corresponding to the lipids, proteins and DNA can be identified having the largest variability within the data (see Table 1). This illustrates the contribution of the wavenumbers to PC1 and PC2. Thus, the loadings clarify what spectral bands can distinguish biofilm positive and biofilm negative strains.

Moreover, PC-loading presentations constitute a valuable tool for estimating the relative contributions from different molecules present in the sample (Figure 7). Such presentations promise to possibly become a viable technique for interpreting overlapping Raman bands/peaks stemming from proteins, nucleic acids, as well as DNA/RNA complexes. However, further detailed work is needed to develop the potential of this approach, and studies are currently in progress to evaluate data sets obtained using point-by-point technique.

Our previous findings strongly suggest that measurements should be taken after exactly the same time of colony incubation for each set of samples to minimise the effect of timing on the Raman spectra. In order to underline the statements made above, in Figure 4, Figure 5 and Figure 6 we show PCA plots for the four *Candida* strains cultured for 48 (±0.5) h. Using just the two principle components one can clearly separate the clusters of spectra associated with the biofilm-positive and negative strains. Note that the data sets used in Figure 4, Figure 5 and Figure 6 were recorded on three different days where the first and the last data collection were six months apart. Figure 6 shows all data obtained from the three measurements which were performed in July 2013, in December 2013 and in February 2014 (not shown in separate plot) in a 3-D plot using PC1, PC2 and PC4 components. Evidently, these three components seem to be sufficient to contain all variations in the two clear clusters.

Not surprisingly, some scatter in the clustering for a given species is observed, suggesting variance within the yeast colonies. However, the observed variance is rather small—clustering of biofilm-positive and negative strains has completely separated the data. We would like to note that any culture will exhibit a certain amount of heterogeneity, and data might be slightly different for spectra associated with points at the edge of the measurement area. Thus, the spread in clustering is associated with the variability in the measurement of a biological sample and carries the requirement for multiple measurements to define a bacterial/yeast population within a species or strain. These findings are consistent with the research of Choo-Smith and co-workers who observed heterogeneity in micro-colony analysis [13].

Inspecting above mentioned figures further, it is remarkable that the clustering of the two data sets (biofilm positive and biofilm negative) shown for the four *Candida* strains, is nearly identical although they were recorded months apart. This demonstrates solid reproducibility in the Raman fingerprints of these *Candida* strains. Clearly this then puts a certain limit onto the time window during which one might be able to directly compare measurements for strain classification. As was mentioned, our previous experiments (not shown here) suggest that this measurement time window is at the most about 1 h, unless one accepts a less accurate overall region into which strain sample data have to cluster. That is why, we investigated our samples always after 48 h of cultivation.

## 3. Species Selection, Sample Preparation and Instrumentation

Candidaemia and invasive candidiasis significantly contribute to the mortality and morbidity of critically ill patients at intensive care units [15,16]. Although *Candida albicans* is the most frequently isolated speciemen from clinical material, the number of non-albicans isolates has dramatically increased in recent years [17,18].

*Candida parapsilosis* is a common part of human microflora—it is often isolated from skin, particularly subungal space of healthy individuals. However, it may act as an opportunistic agent causing nosocomial infections. Besides common infections like paronychia or infections of inner ear it can cause serious invasive infections such as endocarditis or bloodstream infections often leading to sepsis and death of the patient [17,19]. Although the most common source of infection still remains patient himself, hands of healthcare workers may also play the role in a transmission [20].

Patients most susceptible to *C. parapsilosis* infections are very-low-birthweight infants in neonatal intensive care units. Another high risk group are immunocompromised patients often requiring central venous catheters, cannulas, having other foreign prosthetic material in their body (indwelling central venous catheter or other bloodstream implants, heart valves, joint prostheses, *etc.*) as well as undergoing prolonged broad-spectrum antibiotic treatment. [17,21].

The most important issue concerning the pathogenic potential and antifungal resistance of *C. parapsilosis* is biofilm formation. Ability to form strong, adherent layers enables the yeasts to colonise both native and artificial surfaces in the body [22]. It protects them from response of the host immunity system, too. Biofilm positive strains are more resistant to antifungal therapy [23] and as it was reported, they are associated with significantly higher mortality rates of patients with candidaemia than strains incapable of biofilm production [24]. Antifungal therapy alone is insufficient for the cure in this case; affected devices often need to be removed [25]. Therefore, detection of this virulence factor in a particular strain should help to choose an adequate therapy and to assess the prognosis of the patient [17].

### 3.1. Sample Preparation

Two biofilm-positive and two biofilm-negative *Candida parapsilosis* strains (Table 2) were included in the study. All of them were isolated from blood-cultures of patients hospitalised at St. Anne’s Faculty Hospital in Brno [23,26]. The yeast strains included in this study were stored at −70 °C. Before each experiment, the strains were thawed quickly at 37 °C and cultivated on the Mueller-Hinton (MH) agar (Oxoid, Basingstoke, UK) at 37 °C for 48 h.

**Table 2:** Biofilm-positive and negative *Candida parapsilosis* strains [23,26].

Sample Name	Biofilm Positive/Negative
BC 11	+
BC 16	+
BC 45	−
BC 90	−

For biofilm formation testing of *Candida parapsilosis* strains we used modified adhesion assay described by Ruzicka *et al.* [26]. A 48 h yeast culture from Sabouraud dextrose agar (Merck, Schwalbach, Germany) was resuspended in sterile physiological saline solution to the suspension with optical density corresponding to 1 of the McFarland scale. Wells of a 96-well flat-bottomed polystyrene tissue culture microtiter plate (Nunc-Thermo Fisher Scientific, Rosklide, Denmark) were inoculated with 20 μL of the suspension and 180 μL of Yeast Nitrogen Base medium (Difco, Becton, Dickinson and Co., Franklin Lakes, NJ, USA) containing 50 mM glucose and incubated at 37 °C for 24 h. The negative control wells were filled with sterile medium. After incubation, wells were washed and a biofilm layer on the wall and bottom of the wells was fixed by air drying. The adherent biofilm layer was stained with 1% crystal violet for 20 min, washed and air-dried. The bound dye was eluted with 200 μL 33% acetic acid per well and 100 μL of the eluate from each well was transferred to new sterile 96-well flat-bottomed polystyrene tissue culture microtiter plate (Nunc-Thermo Fisher Scientific) for spectrophotometric assessment. Absorbance (A_595_) of each well was measured using Multiscan EX, (A.A. Lab-Systems, Ramat-Gan, Israel) reader. The measurement was performed thrice in 3 wells for each strain. Biofilm positive were considered those wells A_595_ of which was higher than the mean A_595_ of negative controls plus 3× Standard Deviation (SD).

### 3.2. Experimental Setup

**Figure 8:**
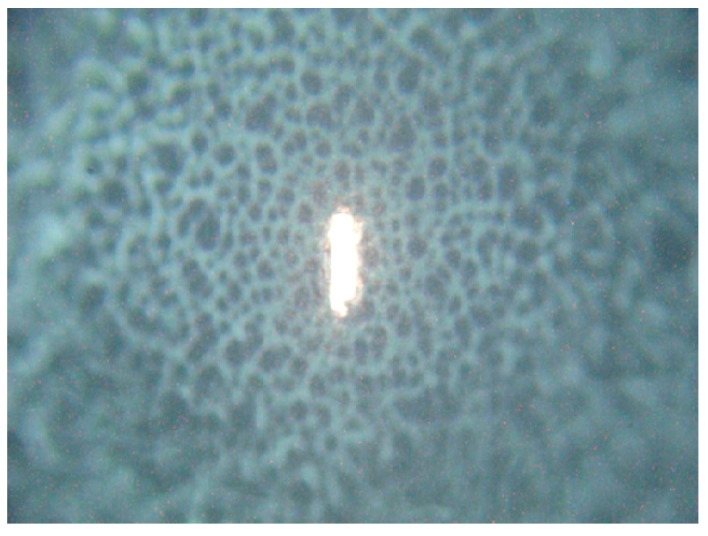
Excitation laser focused by 50× objective onto the central part of the *C. parapsilosis* colony. Note the steep decrease in colony height outside the flat center of growing colony visualized by unfocused colony surface. The vertical size of the laser spot is about 20 µm.

The setup used for Raman microspectroscopy is commercial Renishaw Raman spectrometer (Renishaw inVia Raman Spectrometer, Renishaw plc., Wotton-under-Edge, UK), with 785 nm single-mode diode laser as the excitation source. In our experiments laser beam was focused onto the sample by microscope objective (Leica, Wetzlar, Germany, 50×, NA (Numerical aperture) 0.5), laser spot diameter was about 2 µm × 10 µm (note that such laser spot shape is characteristic for the Renishaw inVia instrument). For simulation of real environment (authentic formation of microorganism) laser was focused onto a surface of *Candida* colony, so we measured response of a small fraction of the colony (see Figure 8) directly on MH agar in Petri dishes.

Overview spectra were acquired in the range of 600–1700 cm^−1^. Each spectrum was measured 30 s from different part of a colony. The Raman spectra were treated with a Savitzky-Golay coupled advanced rolling filter background removal routine (see, e.g., [11]), and then analyzed using a standard multivariate principle component program written in-house using MatLab software (MathWorks, Natick, MA, USA).

## 4. Conclusions

In general, we performed repeated/control measurements for the selected *Candida* strains on colonies grown on the MH agar separated by a given time intervals. These resulted in clusters coinciding well with the biofilm-positive and biofilm-negative strains measurement of a particular sample dish, suggesting good reproducibility of our measurement procedure, even when the samples were prepared and measured days up to months apart. Of course, this holds only true if the preparation, the cultivation, the storage-until-measurement and the Raman analysis were kept within the pre-specified parameter range.

Thus, in principle, the methodology is deemed sufficiently good to conclude that the measurement and evaluation procedure exploited here might well lend itself for reliable diagnostics.
